# Dataset on flow diversion procedures performed with the Pipeline Embolization Device, Pipeline Flex, and Surpass Streamline for intracranial aneurysms

**DOI:** 10.1016/j.dib.2022.108299

**Published:** 2022-05-21

**Authors:** Juan Vivanco-Suarez, Chaim Feigen, Kainaat Javed, Joseph M. Dardick, Ryan Holland, Alan Mendez-Ruiz, Santiago Ortega-Gutierrez, Neil Haranhalli, David J. Altschul

**Affiliations:** a Department of Neurology, Neurosurgery and Radiology, The University of Iowa Hospitals and Clinics, 200 Hawkins Dr, Iowa City, IA 52242, United States; b Department of Neurological Surgery, Montefiore Medical Center, 3316 Rochambeau Avenue, Bronx, NY 10467, United States

**Keywords:** Aneurysm, Flow diversion, Pipeline embolization, Stroke, Subarachnoid hemorrhage, cICA, cavernous portion of the internal carotid artery, DSA, digital subtraction angiography, PED, Pipeline Embolization Device

## Abstract

Flow diversion is an evolving endovascular modality for treating intracranial aneurysms. Although rare, serious adverse events following flow diversion may include ischemic stroke, intracranial hemorrhage, or delayed rupture of the treated aneurysm. This dataset describes 141 flow diversion procedures performed with the Pipeline Embolization Device, Pipeline Flex, or Surpass Streamline on 126 subjects with intracranial aneurysms [1]. The retrospective data were collected from electronic medical records at two large tertiary centers. Baseline patient data included age, sex, and medical comorbidities. The dataset also describes aneurysm characteristics including laterality, anatomic location, morphology, dome height, and neck width. In addition, digital subtraction images showing the internal carotid artery tortuosity were included for aneurysms in the anterior cerebral circulation [2]. Procedural data include case duration, radiation exposure, number of flow diverters deployed, and complications encountered during deployment. In addition, data related to the duration of hospitalization and postoperative adverse events are included. Finally, time to follow up and rates of total aneurysm obliteration at first and second postoperative visits are included. This data is propensity score matching are included. This data is presented as a starting point for future prospective comparisons in the safety and efficacy of flow diverters as more devices become approved and commercially available.

## Specifications Table

**Table:** 

Subject	Surgery
Specific subject area	Flow diversion procedures performed with Pipeline Embolization Device, Pipeline Flex, and Surpass Streamline for the treatment of intracranial aneurysms.
Type of data	Pre-processed data in Excel file, R language script in Word file, library of angiographic images in .jpg format in PowerPoint file
How the data were acquired	The data were extracted by the authors from the electronic medical records at the two centers.
Data format	Raw, de-identified
Description of data collection	Patients who underwent a flow diversion procedure with Pipeline Embolization Device, Pipeline Flex, or Surpass Streamline from October 2012 to February 2020 were included in the data. Demographic data including age, sex, comorbidities, and history of previous aneurysm treatment were collected. Aneurysm laterality, location, morphology, dome height, and neck width were recorded from digital subtraction angiography. For aneurysms located in the cavernous portion of the internal carotid artery (cICA), digital subtraction angiography images were extracted to assess cICA tortuosity using a I-IV grading scale.14 Two authors (C.F. and J.V.-S.) independently reviewed and classified the tortuosity of each cICA case, with each blinded to the other's assessments. Any discordant grading between these authors was resolved by a third author (R.H.), who acted as a tiebreaker and determined the final tortuosity grade. Procedural details including case duration, radiation exposure, and the number of flow diverters deployed were extracted from the operative notes. Technical metrics of the procedures included the success of device deployment, foreshortening, kinking, or adjuvant device use. Periprocedural adverse incidents occurring up to when the patient was discharged from the FD procedure were recorded as well.
Data source location	Institution I: Montefiore Medical Center, Bronx, New York, United States.Institution II: The University of Iowa Hospitals and Clinics, Iowa City, Iowa, United States.
Data accessibility	Repository name: Mendeley Data. Direct URL to data: https://data.mendeley.com/datasets/nzzx92ky6r/2DOI: 10.17632/nzzx92ky6r.2
Related research article	C.M. Feigen, J. Vivanco-Suarez, K. Javed, J.M. Dardick, R. Holland, A. Mendez-Ruiz, S. Ortega-Gutierrez, N. Haranhalli, D.J. Altschul, Pipeline Embolization Device and Pipeline Flex vs Surpass Streamline Flow Diversion in Intracranial Aneurysms: A Retrospective Propensity-Score Matched Study, World Neurosurg. (2022). *In Press,*https://doi.org/10.1016/j.wneu.2022.02.025

## Value of the Data

• Operators with access to Pipeline Embolization Device, Pipeline Flex and Surpass Streamline would benefit from this dataset describing the rates of success and complications for the two classes of flow diverters. These comparative data, as well as previous evaluations of the safety and efficacy of the individual devices, would assist operators in making informed clinical decisions regarding the device selection to treat intracranial aneurysms.
• These data could be combined with other retrospective datasets comparing the same devices for future meta-analyses.
• Our data contains subgroups that can be extracted for further analysis. In addition to comparing cases by device type, this dataset categorizes the indexed aneurysms as ruptured versus unruptured, anterior versus posterior cerebral circulation, parent vessel tortuosity, and fusiform versus saccular morphology.

## 1 Data Description

Following the Pipeline Embolization Device (PED) for the Intracranial Treatment of Aneurysms trial, the field of flow diversion has continuously evolved [3]. Since then, newer generations of devices such as the Pipeline Flex (Covidien, California, USA), Pipeline Flex with Shield Technology (Covidien), Surpass Streamline (Stryker Neurovascular, California, USA), Surpass Evolve (Stryker), and Flow Redirection Endoluminal Device (MicroVention, California, USA) have been developed and approved. Although the mechanism of aneurysm occlusion is similar between devices, their design and delivery system can be considerably different. Since their approval, several articles have demonstrated an encouraging safety and efficacy profile. However, there is limited data that allows side-by-side comparison between devices [4], [5], [6], [7].

This article includes data from flow diversion procedures performed with PED, Pipeline Flex, and Surpass Streamline for the treatment of intracranial aneurysms [1]. The data were collected by the authors from the participating centers (Montefiore Medical Center and The University of Iowa Hospitals and Clinics) into a standardized dataset that contains numerical and continuous data.

**Complete_FD Database.xlsx** file contains the complete raw data. Each subject is assigned a de-identified index value. Pipeline (PED or Pipeline Flex) and Surpass Streamline cases are designated as PLXX and SSXX, respectively. The spreadsheet is divided into three pages: (1) “Demographics” describes baseline patient characteristics and comorbidity variables including age (Age), gender (Gender), previous coronary artery disease (CAD), history of transient ischemic attack, or cerebrovascular accident (TIA/CVA), history of cardiac arrhythmia (Arrhythmia), history of hypertension (HTN), history of hyperlipidemia (HLD), history of diabetes mellitus (DM), history of past or current cocaine (Cocaine), tobacco (Smoker), or alcohol (EtOH) use, history of any cancer (Malignancy). Aneurysm characteristics include laterality (Laterality), specific vessel location (Segment), rupture status (Rupture), size in millimeters (measured on 3D reconstruction) of the dome (Dome Height), neck (Neck width), largest dimension in any direction, and dome-to-neck ratio (DH: NW ratio). Flow diverter dimensions in mm including length (FD Length) and diameter (FD diameter). (2) “Case & Periop metrics” describe the procedural metrics including internal carotid artery cavernous segment tortuosity classification (cICA), success of device implantation (Deployed successfully), procedure (Procedure time) and radiation exposure duration (Fluoro time) in minutes, number of devices deployed (FDs deployed), flow diverter length (FD Length), and diameter (FD diameter) in millimeters. Postprocedural data include subject mortality (Mortality) and the occurrence of new ischemic stroke (Stroke), new intracranial hemorrhage (ICH), retroperitoneal hematoma (RPH), groin hematoma (Groin Hematoma), radial hematoma (Radial Hematoma), carotid dissection (Carotid Dissection), and corneal abrasion (Corneal abrasian). Hospitalization characteristics include new urinary tract infection (UTI), pneumonia (PNA), deep venous thrombosis (DVT), length of hospital stay (LOS) in days, and type of patient disposition (Dispo). (3) “FU” depicts the months to first (Months to FU 1) and second (Months to FU 2) digital subtraction angiography (DSA) follow-up along with a binary result indicating whether the angiogram demonstrated complete aneurysm obliteration (FU1 100% occluded?).

**R script for Propensity score matching.docx** contains the detailed R script used to perform the propensity score matching.

**cICA_PipevsSurpass.pptx** contains the DSA images in .jpg format of the cICA of the patients with anterior circulation aneurysms. These images were used to assess the tortuosity of the cICA using a I-IV grading scale [2].

## 2 Experimental Design, Materials and Methods

### 2.1 Inclusion Criteria

Subjects included for the data collection were patients who underwent a flow diversion procedure with Pipeline Embolization Device, Pipeline Flex, or Surpass Streamline from October 2012 to February 2020 at two large academic centers.

### 2.2 Data Collection Strategy

A dataset and data dictionary of variables was created by the coordinating center (Montefiore Medical Center) author (C.F.) and shared with the collaborating center (The University of Iowa Hospitals and Clinics) for consensus and standardization. After universal agreement on the variables and outcome definitions, each participating center collected the predefined clinical and radiographic variables. The data was extracted from the electronic medical records from each included subject. After complete dataset collection, the file was shared with the coordinating center through a secured web-based portal.

### 2.3 Baseline Subject and Imaging Characteristics Data Collection

**Fig. 1:**
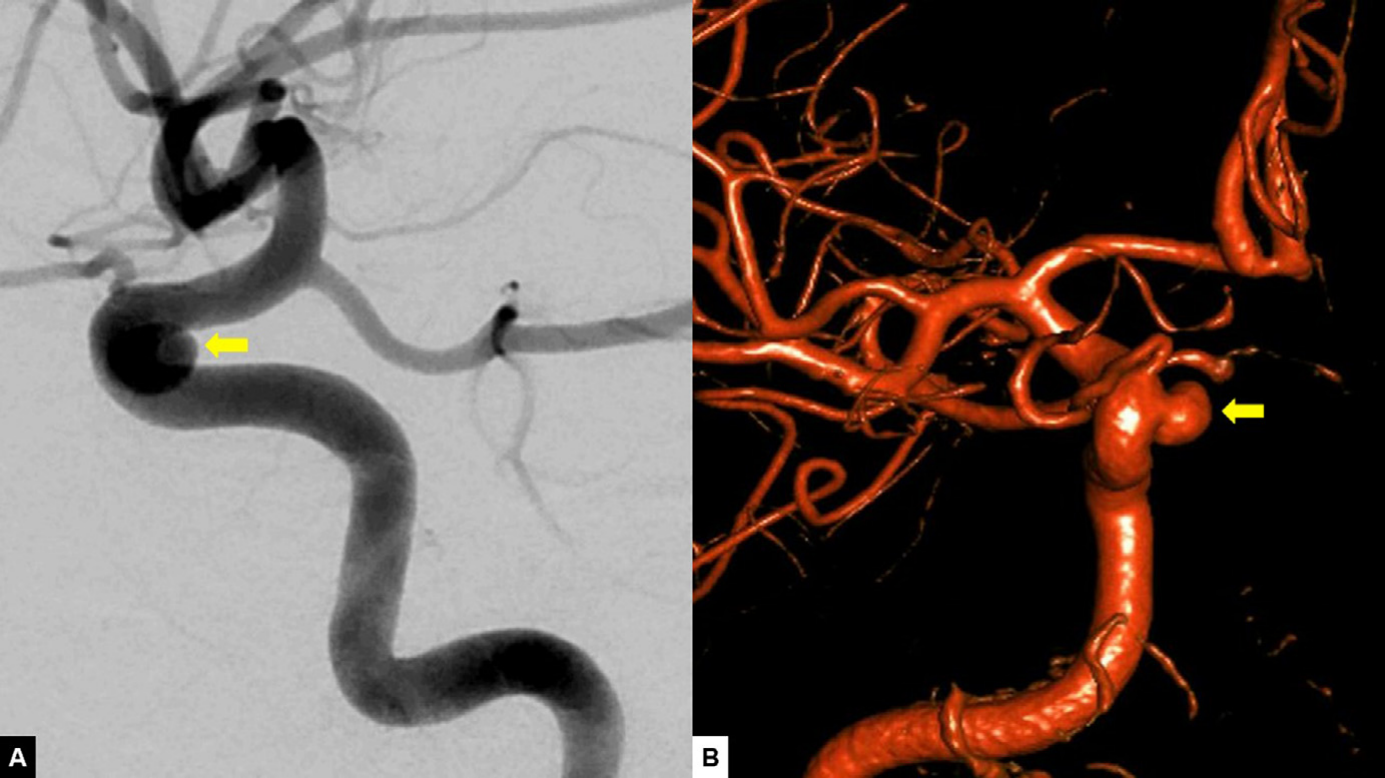
Aneurysm characteristics example. This is a case of a 63-year-old female with a history of hypertension who was found to have a 6.0 × 5.5 mm saccular aneurysm located in the paraophthalmic segment of the right internal carotid artery found incidentally during work-up for acute, severe headache. Flow diversion was achieved using a 3 × 20 mm Surpass Streamline. (A) Digital subtraction angiography showing a lateral view of the right internal carotid artery. The yellow arrow points to the saccular aneurysm in the paraophthalmic segment. (B) Anterior-posterior view of 3D-reconstructed digital subtraction angiography. The yellow arrow points to the saccular aneurysm, with the dome projecting medially.

**Fig. 2:**
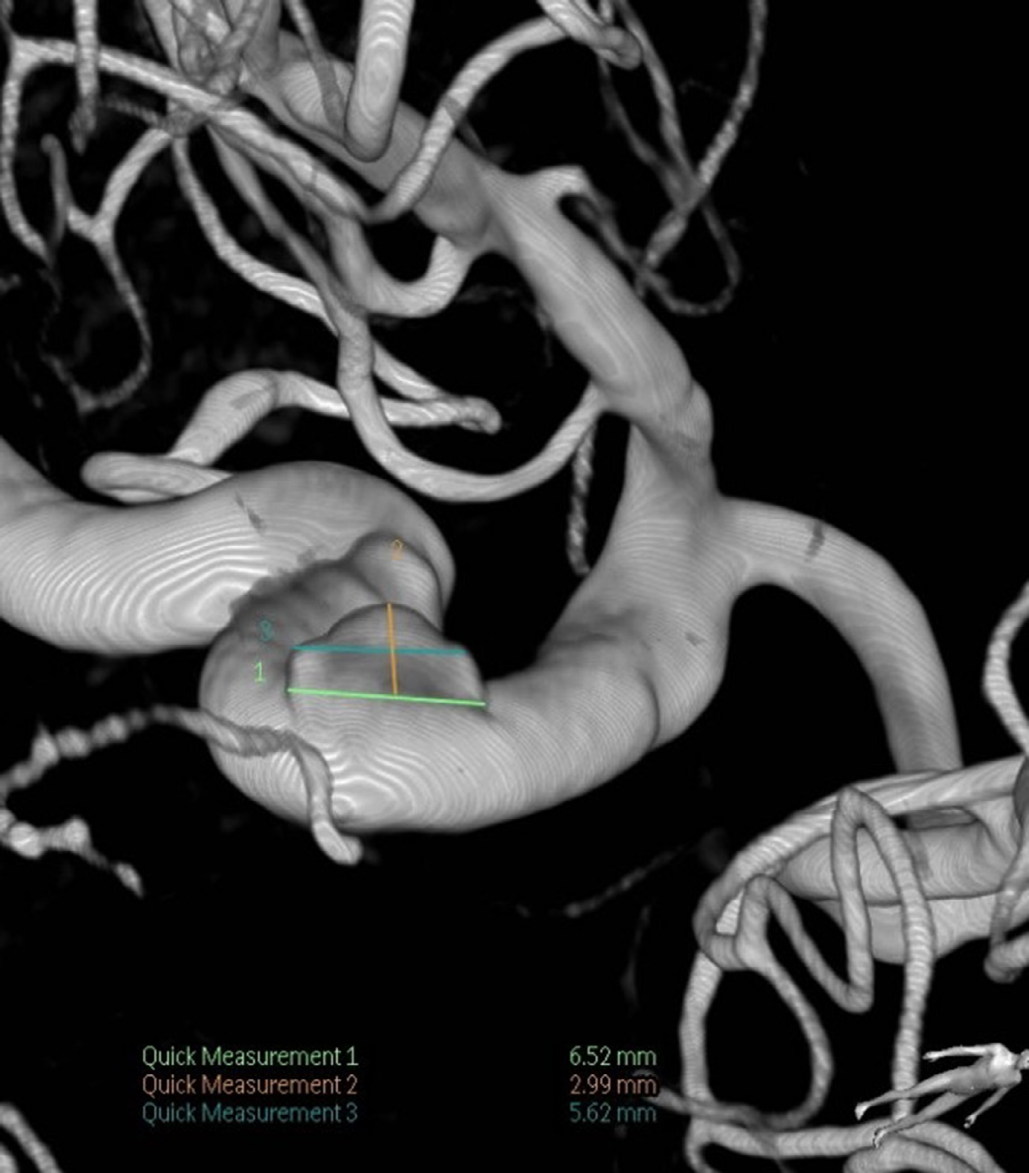
Aneurysm dimension measurement example. This is a case of a 70-year-old female with a history of hypertension, hyperlipidemia, smoking, and type 2 diabetes mellitus in whom a bilobed 3.0 × 5.6 mm aneurysm was identified in the cavernous segment of the right internal carotid artery after a syncopal episode. Flow diversion was achieved using a 5 × 12 mm Pipeline Embolization Device. The image shows the anterior-posterior view of a 3D reconstruction of digital subtraction angiography. The measurement of the aneurysm height (orange line and text), dome width (light green line and text), and neck width (dark green line and text) are displayed.

The initial clinical patient visit before the aneurysm flow diversion procedure was thoroughly reviewed to collect the subject's age, gender, history of coronary artery disease, history of transient ischemic attack or cerebrovascular accident, history of cardiac arrhythmia, history of hypertension, history of hyperlipidemia, history of diabetes mellitus, history of past or current cocaine, tobacco, or alcohol use, and history of any malignancy. The aneurysm characteristics were obtained from the pretreatment DSA and/or 3D reconstruction (Fig. 1). The 3D reconstructions used a vessel enhancing kernel and a smooth image impression focused to minimize image noise [8]. Aneurysm's characteristics include laterality, specific anatomic location, and morphology. The aneurysm dimensions (in millimeters) include dome height, neck width, largest aneurysm dimension, and dome-to-neck ratio. The dome height was measured perpendicular to the neck. The neck width was measured at the widest point of the aneurysm neck. Measurements were performed in the 3D reconstruction images by the treating neurointerventionalist (Fig. 2). In specific cases, aneurysm measurements were not described or recorded in the initial angiographic studies. For those cases, the 3D reconstruction images were reviewed and measurements were obtained by one of the authors. The previously described method of measurement was applied. In addition, for aneurysms located in the anterior circulation, the tortuosity of the cavernous segment of the internal carotid artery (cICA) was assessed using a I-IV grading scale described by Lin et al. [2]. Two independent investigators (C.F. and J.V.-S.) not involved in the treatments reviewed DSA images to classify the tortuosity of the cICA. When disagreement in the classification occurred, the discrepancy was resolved by a third investigator (R.H.).

### 2.4 Procedural and Postprocedural Data Collection

Procedural metrics describe the success of device implantation, procedure and radiation exposure duration in minutes, number of devices deployed, flow diverter length, and diameter (FD diameter) in millimeters. All the data was obtained from a review of the procedure note written by the treating neurointervenionalist.

Postprocedural data collection started after the completion of the target aneurysm treatment. This data was obtained from the daily clinical and discharge notes. It includes subject mortality and the occurrence of new ischemic stroke, new intracranial hemorrhage, retroperitoneal hematoma, groin hematoma, radial hematoma, carotid dissection, and corneal abrasion. In addition, hospitalization characteristics including length of hospital stay in days, new urinary tract infection, a new diagnosis of pneumonia, new deep venous thrombosis, and type of patient disposition, were reviewed.

### 2.5 Follow-up Data Collection

The follow-up data collection was performed at different time points according to the treating center protocols and the patients. The electronic medical record of each subject was reviewed for the first and second follow-up clinic visits and the associated imaging studies performed. The time in months between the procedure and the visit was calculated. The imaging data was assessed considering a binary result indicating whether the aneurysm was completely obliterated or not.

### 2.6 Statistical Analysis

The propensity score matching R scripts included in the data construct a logistic regression model with group 1 versus group 2 considering the outcome variable of interest. The covariates selected are used to generate propensity scores for each of the subjects. To perform a “Nearest-neighbor” matching the R package MatchIt (version 3.6.0; PBC, Boston, Massachusetts, USA) assigs matched pairs of group 1 and group 2 based on propensity score assignments. This model allows one to design and analyze an observational study so that it creates the scenario characteristic of a randomized control trial to estimate the impact of an intervention [9].

## Ethics Statements

This data collection was approved by the institutional review boards for both centers, and informed consent was waived due to the observational nature of the data collection.

Montefiore Medical Center IRB #2019-10214

The University of Iowa Hospitals and Clinics IRB #2020-05349

## CRediT authorship contribution statement

**Juan Vivanco-Suarez:** Conceptualization, Methodology, Software, Formal analysis, Investigation, Resources, Data curation, Writing – original draft, Writing – review & editing, Visualization, Project administration. **Chaim Feigen:** Methodology, Formal analysis, Data curation, Writing – review & editing, Visualization. **Kainaat Javed:** Formal analysis, Writing – original draft. **Joseph M. Dardick:** Writing – original draft, Writing – review & editing. **Ryan Holland:** Writing – original draft, Writing – review & editing. **Alan Mendez-Ruiz:** Resources, Data curation. **Santiago Ortega-Gutierrez:** Writing – review & editing, Supervision. **Neil Haranhalli:** Conceptualization, Writing – original draft, Writing – review & editing, Supervision. **David J. Altschul:** Conceptualization, Data curation, Writing – original draft, Writing – review & editing, Supervision.

## Declaration of Competing Interest

The authors declare that they have no known competing financial interests or personal relationships that could have appeared to influence the work reported in this paper.

## Data Availability

Data from Pipeline Vs Surpass Flow Diversion Study (Original data) (Mendeley Data).
